# Combined Neuroprotective Strategies Blocked Neurodegeneration and Improved Brain Function in Senescence-Accelerated Mice

**DOI:** 10.3389/fnagi.2021.681498

**Published:** 2021-08-23

**Authors:** Helena Nascimento Malerba, Arthur Antonio Ruiz Pereira, Marcela Favoretto Pierrobon, Guilherme Souza Abrao, Mariana Toricelli, Eliana Hiromi Akamine, Hudson Sousa Buck, Tania Araujo Viel

**Affiliations:** ^1^Graduate Course on Pharmacology, Institute of Biomedical Sciences, Universidade de São Paulo, São Paulo, Brazil; ^2^Laboratory of Neuropharmacology of Aging, School of Arts, Sciences and Humanities, Universidade de São Paulo, São Paulo, Brazil; ^3^Department of Physiological Sciences, Santa Casa de São Paulo School of Medical Sciences, São Paulo, Brazil

**Keywords:** active life expectancy, enriched environment, lithium carbonate, senile plaques, neuroprotection, healthy aging, SAMP-8

## Abstract

Increase in the quality of life, combined with drug strategies, has been studied as possibilities for improving memory and delaying the onset of neurodegenerative diseases. A previous study published by the group of the authors has shown that microdose lithium and enriched environment can improve memory in both mice and humans. To elucidate this relationship better, this study aimed to evaluate whether the chronic combination of these two strategies could increase healthy aging in Senescence Accelerated Mouse-Prone 8 (SAMP8). Animals were submitted to either one or both of these strategies until the age of 10 months when they were anesthetized and killed and their hippocampus was extracted. The untreated SAMP-8 group exhibited worse memory and reduced neuronal density with greater neurodegeneration and increased amyloid-β plaque density compared with the control group. Moreover, significant alterations in proteins related to long-term potentiation, such as, synaptophysin and brain-derived neurotrophic factor (BDNF), were observed in this group. The strategies used in the study maintained long-term memory, reduced anxiety, and increased neuroprotection. Both strategies were efficient in reducing neurodegeneration and increasing parameters related to memory maintenance. In many experiments, the combination of the two strategies was more effective in improving healthy aging. This study sheds light on the combination of strategies that choose to improve the quality of life and drugs with low side effects. Moreover, it opens perspectives for a new field of study for healthy aging.

## Highlights

- SAMP-8 is a mouse model of accelerated aging and Alzheimer's disease.
- SAMP-8 presents spatial memory deficits and anxious behavior.
- Animals were submitted to an enriched environment and/or treatment with lithium.
- Combined strategies blocked neurodegeneration and maintained neuroplasticity.
- Data give support to the use of complementary strategies to improve healthy aging.

## Introduction

It is already known that increased longevity gives rise to chronic diseases that can be highly debilitating and influence the quality of life, as is the case of Alzheimer's disease (AD). AD is a major cognitive disorder associated with amyloid-β (Aβ) deposits and the formation of neurofibrillary tangles leading to neuronal degeneration and the failure of mechanisms related to the formation, retention, and recovery of memory (Ballard et al., 2011; Peña-Ortega and Bernal-Pedraza, 2012).

Learning and memory formation involve a biological process known as long-term potentiation (LTP) that increases dendritic branches and interaction among neurons leading to consolidation of information (Valenzuela, 2008; Petrosini et al., 2009; Balthazar et al., 2018). LTP happens because of the synaptic activation of n-methyl-d-aspartate (NMDA) and α-Amino-3-hydroxy-5-methyl-4-isoxazolepropionic acid (AMPA) glutamate receptors (Bliss and Collingridge, 1993). The activation of these receptors leads to an increase in intracellular calcium and activation of kinase proteins (Nicoll, 2017). In neurons, the activation of calcium-calmodulin dependent protein kinase IV promotes the activation of the transcription factor cAMP-response element-binding protein (CREB) through phosphorylation (Finkbeiner, 2000; Mayr and Montminy, 2001; Kokubo et al., 2009), which increases neurotrophin synthesis. Brain-derived neurotrophic factor (BDNF) is one of these neurotrophins, and it plays a fundamental role in the consolidation of LTP (Finkbeiner, 2000; Mayr and Montminy, 2001; Kokubo et al., 2009).

Among NMDA receptor subtypes, NMDA-R2B is essential for synaptic stabilization, mainly during neuronal maturation (Groc et al., 2009). Recent studies have shown that this receptor is responsible for the increase in excitotoxicity in patients with AD, as the great increase in Aβ plaques in the hippocampus leads to substitution of the Glu-N2A subunit with Glu-N2B. This allows the receptor to migrate to extra-synaptic regions and be combined with the SAP-102 receptor, avoiding the internalization of Glu-N2B. The activation of this receptor in extra-synaptic regions results in neuronal death through apoptosis (Hardingham and Bading, 2010; Parsons and Raymond, 2014; Bading, 2017).

These events, combined with others that decrease neuronal structure and strength, lead to memory loss that is so common in older adults. Therefore, the formation of a brain reserve (both structural and cognitive) through alterations in daily habits can effectively change brain aging and may be an important strategy for memory retention, as has been demonstrated in several studies (Balthazar et al., 2018; Merrill et al., 2019; Mohammadian et al., 2019).

Many strategies, both pharmacological and non-pharmacological, have been used to promote cognitive and structural reserves. Among them, lifestyle changes, also known as environmental enrichment, is widely known to promote memory preservation and decrease Aβ plaque in animal models of AD (Jankowsky et al., 2005; Balthazar et al., 2018), while also increasing the production of BDNF (Baraldi et al., 2013; Dong et al., 2018; Kaptan et al., 2019), suggesting that improvements in cognitive performance and neurogenesis are related to a more active life (Garthe et al., 2016).

Recently, parallel to the effects of an enriched environment, the research team demonstrated the promising use of the administration of microdoses of lithium in neurodegenerative processes linked to Alzheimer's disease (AD). Classically, it is reported that lithium decreases oxidative stress and the activity of GSK-3β (Chalecka-Franaszek and Chuang, 1999), reducing the formation of the toxic oligomers of Aβ while increasing the expression of anti-apoptotic genes such as Bcl-2 (O'Leary et al., 2012), as well as BDNF expression (Forlenza et al., 2012, 2019), promoting neuroprotection. When used in microdoses, lithium can promote memory preservation and decrease anxiety (Nunes et al., 2015) and neuroinflammation (Toricelli et al., 2020), and it also exhibits senolytic properties (Viel et al., 2020), verified both in AD animal models and in human-derived induced pluripotent stem cell (iPSCs) astrocytes.

This study aimed to evaluate whether the combined use of microdose lithium and enriched environment could have a greater benefit for healthy brain aging compared with the use of these strategies separately.

## Materials and Methods

### Animals

The animals used in this study were derived from AKR/J mice. Breeding between siblings originated offspring with some different characteristics from the matrices. The animals with those characteristics were selected and classified as having a propensity to accelerate aging and were named as series P. The other animals that presented normal aging were selected as matrices of a more resistant mice strain was and were named series R. In this way, mice with accelerated aging were named SAMP (“senescence-accelerated mice prone”) and resistant mice were named SAMR (“senescence-accelerated mice resistant”) (Takeda et al., 1997). Analyzing the characteristic of each strain, for this study, SAMP-8 animals were selected. Besides the characteristic of accelerated aging, their life expectancy is about 10 months, and they show learning and memory deficits, early amyloid-β deposition in the hippocampus, blood-brain barrier dysfunction, cortical atrophy, and neuronal loss, among other features. In this way, this is a promising model for the study of neurodegenerative conditions, such as AD (Takeda, 1999).

**Figure 1:**
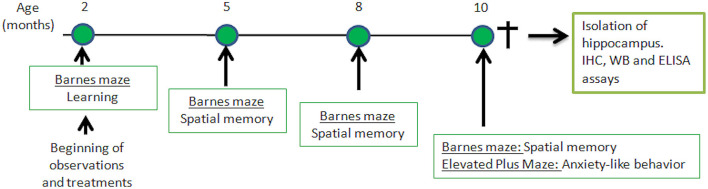
Time-line of behavioral experiments and further analysis of hippocampus isolated from senescence-accelerated mice resistant (SAMR)-1 and Senescence Accelerated Mouse-Prone 8 (SAMP-8) animals submitted or not submitted to oral microdosing with lithium and enriched environment. Treatments were maintained throughout the lives of the animals, and 10 months of age was the end-point for all treatments.

Senescence-Accelerated Mouse Prone-8 (SAMP-8, *n* = 36) and Senescence-Accelerated Mouse Resistant-1 (SAMR-1, *n* = 10) were provided from the colony, and were maintained in ventilated racks with controlled room temperature (22–24°C) and humidity (55–65%), and food and water *ad libitum*, in a controlled 12 h light/dark cycle. The SAMP-8 animals were divided into the following groups: animals with no treatment (Control, *n* = 10), animals treated with microdoses of lithium (Li, n = 10), animals submitted to an enriched environment (EE, *n* = 8), and animals treated with lithium and submitted to an enriched environment (Li + EE, *n* = 8). Treatments were initiated when the animals were 2 months old and maintained until they reached 10 months old. At this point, the animals were anesthetized and killed by decapitation. The hippocampi were isolated and prepared for histochemical and biochemical analyses. The experimental design is shown in Figure 1.

All the animal procedures were performed according to the 3 R's principle (Replacement, Reduction and Refinement) and the Guide for Care and Use of Laboratory Animals (Bethesda, MD, United States). The experimental protocols were approved by the Animal Ethics Committee of the Santa Casa de São Paulo Medical School (2016-12), Sáo Paulo, Brazil. All efforts were made to minimize the number of animals and their level of suffering.

### Enriched Environment (EE)

After the first spatial memory test, the animals were placed in an EE (when they were 2 months old). To create the EE, standard cages (414 × 344 × 174 mm) were filled with objects that were changed every 2–3 days, as reported before (Baraldi et al., 2013). The objects were made of different materials such as plastic, wood, glass, or aluminum, and included ladders, balls, ropes, small houses, etc. The animals were kept in groups of 4–5 in each cage, and food was sometimes hidden in the bedding. These actions meant that the animals experienced sensorial, physical, and social enrichment.

The animals in standard housing were also placed in standard cages in groups of 4–5, containing only the bedding. All the groups received food and water (with or without lithium) *ad libitum*.

### Treatment With Microdoses of Lithium

Lithium carbonate was dissolved in filtered tap water and offered *ad libitum, per os*, to the animals, resulting in a final dosage of 0.005 mg/animal/day, which can be considered to be in the microdose range as described earlier by the group of the authors (Nunes et al., 2013, 2015; Toricelli et al., 2020, 2021), The cited dosage corresponds to 0.25 mg/kg. The quantity of water consumed by the lithium-treated and untreated groups was not significantly different (average liquid consumption of the untreated groups: 44 ± 0.6 ml/week; average liquid consumption of the treated groups: 44.2 ± 0.9 ml/week).

### Behavioral Tests

#### Evaluation of Spatial Memory

Spatial memory was evaluated using the Barnes maze, following a protocol frequently used in the laboratory of the authors (Baraldi et al., 2013). Briefly, the equipment comprised a white arena (diameter: 100 cm) positioned 1 m from the floor. The arena had 30 holes (each with a 5 cm diameter), disposed radially, and a black cardboard wall around its circumference. The wall had four yellow figures to add spatial orientation, and a fluorescent lamp was placed above the center of the arena. The illumination created an uncomfortable environment that motivated the animals to escape from the arena to a dark box filled with sawdust placed under one of the holes (Baraldi et al., 2013). The learning session was performed when the animals were 2 months old. At this time, each animal was placed in the center of the arena under an acrylic box for 1 min. The animal was then released, and the time it found the escape box was recorded. The maximum time to explore the maze was set to 5 min. The protocol was repeated in the following 5 days. The escape box was placed in the same hole for the same animal, at each testing time, but was different for each animal. The escape box was placed in the right quadrant. The maze was cleaned with a 5% ethanol solution after each animal exposition. The protocol was repeated when the animals turned 5, 8, and 10 months old.

The total time spent to find the escape box and the time spent in the right quadrant (meaning the location of the escape box) were recorded and analyzed in each group at different ages. In addition, comparison among groups at each age was also performed.

#### Evaluation of Anxiolytic-Like Behavior

Anxiolytic-like behavior was evaluated when the animals were 10 months old, using the elevated plus-maze as described in an earlier study (Nunes et al., 2015). Briefly, the animals were placed in the center of the maze, and the free behavior was recorded for 5 min. The time spent in each open arm and each closed arm, and the number of entries in each arm, were registered and presented as the ratio between time spent in the open and closed arms. The experiment was recorded using a JVC Everio (JVC, Kanagawa, Japan) video camera and then analyzed using Smart Software, version 2.5.21 (Panlab Harvard Apparatus). The test was performed when the animals were 10 months old.

### Quantification of Neuronal Density

After behavioral observations, the animals were killed by decapitation, and their brains were extracted. They were immediately frozen in dimethylbutane and stored at −80°C until use. Brain samples (20 μm) were obtained in a cryostat (−20 to −22°C, MicromHM505N, Francheville, France), and the sections were mounted on gelatin-coated slides, desiccated for 5 min at room temperature, and kept at −80°C until use (Baraldi et al., 2013). For neuronal labeling, the slides were warmed to room temperature and incubated with an anti-NeuN primary antibody (ABN78, 1:500, Millipore, Burlington, MA, United States) for 1 h. After that, they were washed with phosphate-buffered saline (PBS) for 5 min and incubated with secondary antibody Alexa Fluor 594 (goat anti-rabbit IgG, 1:200, Life Technologies, Grand Island, NY, United States) for 2 h. After this period, the samples were washed once more with PBS for 5 min and mounted under coverslips with Fluoroshield^TM^ with 4', 6-diamido-2-phenylindole (DAPI) for labeling of the cell nucleus (F6057, Sigma-Aldrich, St. Louis, MO, United States).

Images were obtained using an inverted DMi8 microscope (Leica, Wetzlar, Germany). Quantification was performed using ImageJ software 1.51j8 (National Institutes of Health, Bethesda, MD, United States) in three different hippocampal regions between −1.7 and −2.46 mm from bregma (Franklin and Paxinos, 2007) for each animal.

### Analysis of Senile Plaque Density

To identify and quantify senile plaques, slides containing brain slices were warmed to room temperature and washed with phosphate-buffered saline (PBS) (pH 7.4) containing 1% Triton-X (PBST) for 3 min, three times. The slides were then immersed for 5 min in a Thioflavin S (T1892, Sigma-Aldrich, St. Louis, MO, United States) solution (0.1%) and diluted in PBST. A new sequence of washes with PBST was performed and, after that, the slides were closed with Fluoroshield^TM^ with DAPI (F6057, Sigma-Aldrich, St. Louis, MO, United States) and stored at 4°C. Images of the hippocampus were obtained using an inverted microscope (DMi8, Leica, Wetzlar, Germany). Amyloid-β plaques were quantified in the CA1, CA3, and granular layer of the dentate gyrus (GrDG) of each animal, in the region between −1.7 and −2.46 mm from bregma (Franklin and Paxinos, 2008) using the ImageJ 1.51j8 software (National Institutes of Health, Bethesda, MD, United States).

### Detection of Cells Under Neurodegeneration

Detection of neurodegeneration was performed using Fluoro-Jade C. The slides were dried for 10 min and fixed with 4% paraformaldehyde for 15 min. They were then dipped in 80% alcohol with 1% NaOH for 15 min followed by 70% ethanol for 2 min. After that, the slides were washed in distilled water for 2 min and dipped in 0.06% potassium permanganate for 2 min before being washed with distilled water for 10 min and incubated for 1 h with a Fluoro-Jade C solution for the labeling of neurodegeneration, and DAPI labeling of the cellular nuclei. Following this, the slides were washed three times (1 min) with distilled water. Finally, the slides were dipped twice in xylol and closed with DPX (100909960).

For immunofluorescence labeling, images of the hippocampal regions CA1, CA3, and GrDG between −1.7 and −2.46 mm from bregma were obtained using an inverted DMi8 microscope and analyzed using the LAS X software (Leica Microsystems, Wetzlar, Germany).

### Western-Blot for Detection of Proteins Related to Neuronal Structure and Memory Processing

Hippocampal samples of six animals from each group were isolated and homogenized in a lysis buffer containing 50 mM Tris-HCl (pH 7.4),0.1% Triton X-100, 4 mM EGTA, 10 mM EDTA, and a tablet of protease and phosphatase inhibitors (Roche, Basel, Switzerland). A homogenized tissue was centrifuged at 12,000 rpm for 15 min at 4°C. Protein quantification was performed using the supernatant, following the method of Bradford (Bradford, 1976). Similar quantities of proteins from the homogenate were separated in a polyacrylamide gel (10% SDS-PAGE) and transferred to a nitrocellulose membrane. The unspecific binding was blocked with a 1% Tween-20 Tris buffer containing 2% bovine serum albumin for 1 h at room temperature. After that, the membrane was incubated with Ponceau S for 5 min for labeling of transferred proteins. The membranes were then washed and incubated overnight with primary antibodies for NMDA-2B (ab65783, Abcam, Cambridge, United Kingdom), Glu2/3/4 (2460S, Cell Signaling Technology, Danvers, MA, United States), CAMKIV (4032S, Cell Signaling Technology, Danvers, MA, United States), or synaptophysin (ab8049, Abcam, Cambridge, United Kingdom). On the following day, the membranes were incubated with a secondary antibody (Abcam, ab6721 Rb) for 2 h at room temperature, and protein density was quantified using the ImageJ software (Abramoff et al., 2004).

### Quantification of Total BDNF

Total brain-derived neurotrophic factor (BDNF) was quantified using a commercial Total BDNF Quantikine ELISA kit (DBNT00, R&D Systems, Minneapolis, MN, United States) according to the instructions of the manufacturer.

### Statistical Analysis

Data were expressed as means ± standard error of means and analyzed using GraphPad Prism 5.02. All the data were submitted to the D'Agostino and Pearson omnibus normality test. As long as they passed, a parametric analysis was performed. For comparison between the SAMR-1 and SAMP-8 mice, the Student *t*-test for unpaired data was performed. Behavior data were submitted to two-way ANOVA, followed by Bonferroni's test. For all other data related to isolated or combined treatments of SAMP-8, the data were or submitted to one-way ANOVA followed by Tukey–Kramer test for multiple comparisons. Differences were considered significant when *p* < 0.05.

## Results

### Improvement in Spatial Memory of SAMP-8 Mice

**Figure 2:**
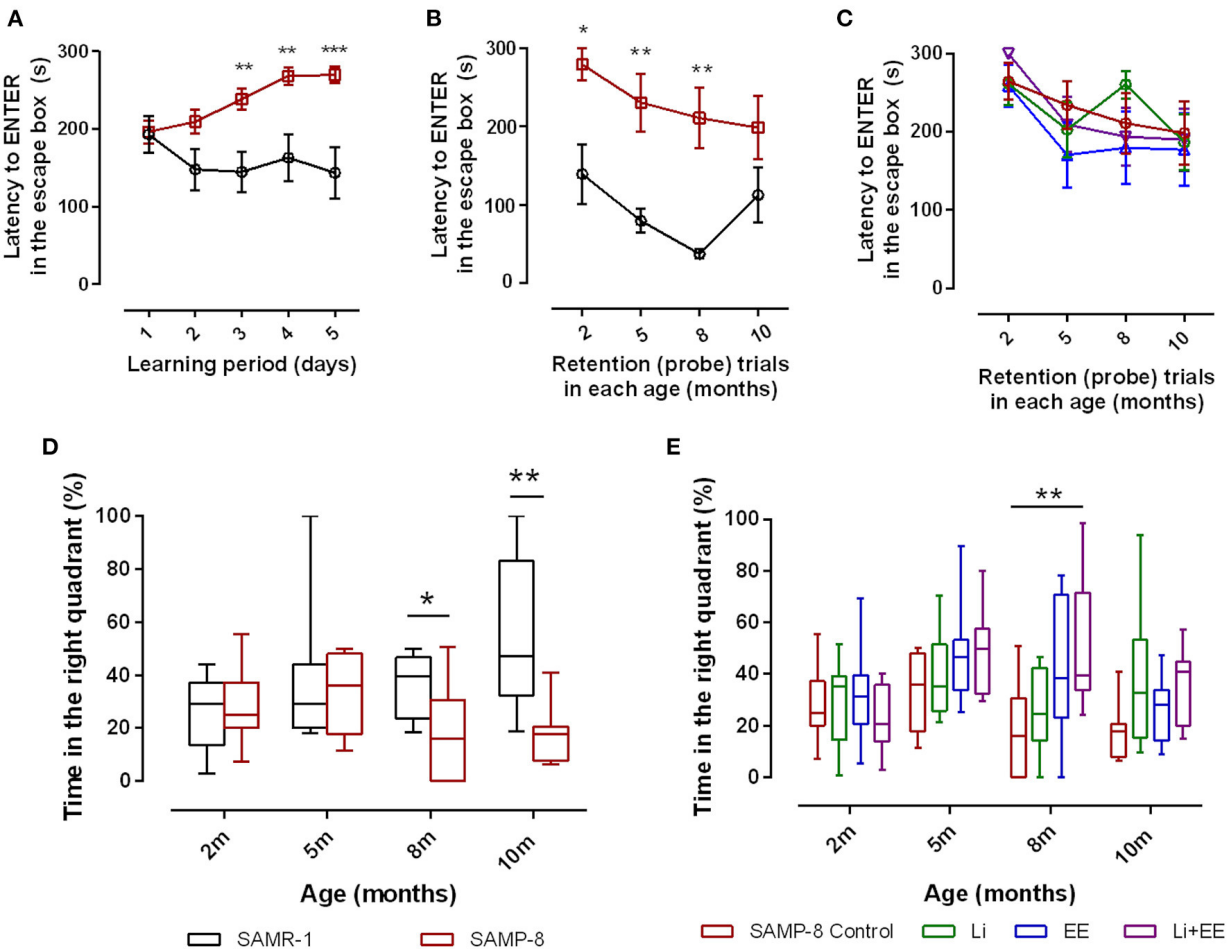
Senescence Accelerated Mouse-Prone 8 (SAMP-8) worse memory was improved with different strategies along with aging. Spatial memory was analyzed using the Barnes maze test. **(A)** Comparison of total time spent in the maze (TT) between the SAMR-1 and SAMP-8 groups during the learning period (5 consecutive days). **(B)** Comparison of TT between the SAMR-1 and SAMP-8 groups during retention (probe) tests between 2 and 10 months of age. **(C)** Comparison of TT among the SAMP-8 animals submitted to different strategies along time. **(D)** Comparison of time spent in the right quadrant between SAMR-1 and SAMP-8 along time. **(E)** Comparison of time spent in the right quadrant among the treated and untreated SAMP-8 groups. Histograms and vertical bars are means ± standard error of the mean (SEM). *p < 0.05; **p < 0.01; ***p < 0.001.

To evaluate the spatial memory of SAMR-1 and SAMP-8, animals were submitted to the Barnes maze for 5 consecutive days (learning period). The total time to find and enter in the escape box (TT) was observed, and SAMR-1 presented a constant TT from days 2 to 5, which means that they learned and kept the memory for the task. Nevertheless, SAMP-8 showed significant increases in TT, evidencing the difference in spatial memory between both groups [*F*_(1,56)_ = 14.91, *p* < 0.001, Figure 2A] The interaction between the factors time and strain was also significant [*F*_(4,224)_ = 4.12, *p* < 0.01]. When submitted to probe trials at 5 and 8 months of age, the TT difference between the groups was maintained, which means that SAMP-8 could not remember the task even with repetition [*F*_(1,59)_ = 33.01, *p* < 0.0001, Figure 2B]. The increase in time to find the escape box of SAMR-1 at 10 months of age was attributed to habituation to the task. When comparing the TT of untreated SAMP-8 with that of SAMP-8 submitted to the strategies, no difference among the groups was observed [*F*_(3,129)_ = 0.7317, *p* = 0.53, Figure 2C]. Together with the TT, the time spent in the right quadrant (the quadrant in which the escape box was placed under the arena in the Barnes maze) was also analyzed. In this way, a significant interaction between the factors time and strain [*F*_(3,52)_ = 3.176, *p* < 0.05] was observed, as the SAMR-1 group significantly increased their time spent in the right quadrant when the animals were re-exposed to the maze (mean difference between time spent at 2 months old and 10 months old = −29.83, *p* < 0.05, Figure 2D). The untreated SAMP-8 group did not have the same behavior, as the time spent in the right quadrant was not altered significantly. In this way, a significant difference between both groups was observed along the aging process [*F*_(1,52)_ = 8.787, *p* < 0.01]. The SAMP-8 group spent significantly less time in the right quadrant at 8 months of age (18.4%, *p* < 0.05) and at 10 months of age (25.5%, *p* < 0.01), when compared with the SAMR-1 group (Figure 2D). The analysis of the behavior of the four SAMP-8 groups (untreated, Li, EE, and Li + EE) showed a significant difference in the factor time [*F*_(3,114)_ = 4.156, *p* < 0.01] and the factor treatments [*F*_(3,114)_ = 3.79, *p* < 0.05], although no interaction between the factors was registered. The animals from the Li + EE group had a significant increase in time spent in the right quadrant when they were 5 and 8 months old (mean difference between 2 months old and 5 months old = −25.9 and −25.5, respectively), but this was not found with respect to the groups that only received one of the treatments (groups Li and EE) (Figure 2E). Also, at 8 months of age, the Li + EE animals showed a significant 2.5-fold increase (*p* < 0.01) in time spent in the right quadrant when compared with the untreated SAMP-8 (Figure 2E). There was no difference in this parameter among the other groups.

For better comprehension of the results, the abundance of time spent in the wrong quadrants by all the SAMP-8 groups is presented in Table 1.

**Table 1:** Percentage of time (mean ± SEM) of permanence of SAMP-8, submitted or not to the different strategies, in wrong quadrants, in the different time points.

	**2 months of age**	**5 months of age**	**8 months of age**	**10 months of age**
SAMP-8 (Control)	70.96 ± 5.25	66.36 ± 5.5 1	73.12 ± 5.61	80.55 ± 4.27
Li	71.52 ± 5.35	61.70 ± 6.39	71.12 ± 7.50	63.67 ± 9.55
EE	67.84 ± 6.58	51.75 ± 6.89	57.26 ± 9.57	73.91 ± 4.41
Li + EE	77.03 ± 4.52	51.13 ± 15.82	50.38 ± 9.11	65.79 ± 5.71

### Enriched Environment Plus Lithium Was Able to Produce Anxiolytic-Like Effects in SAMP-8 Mice

**Figure 3:**
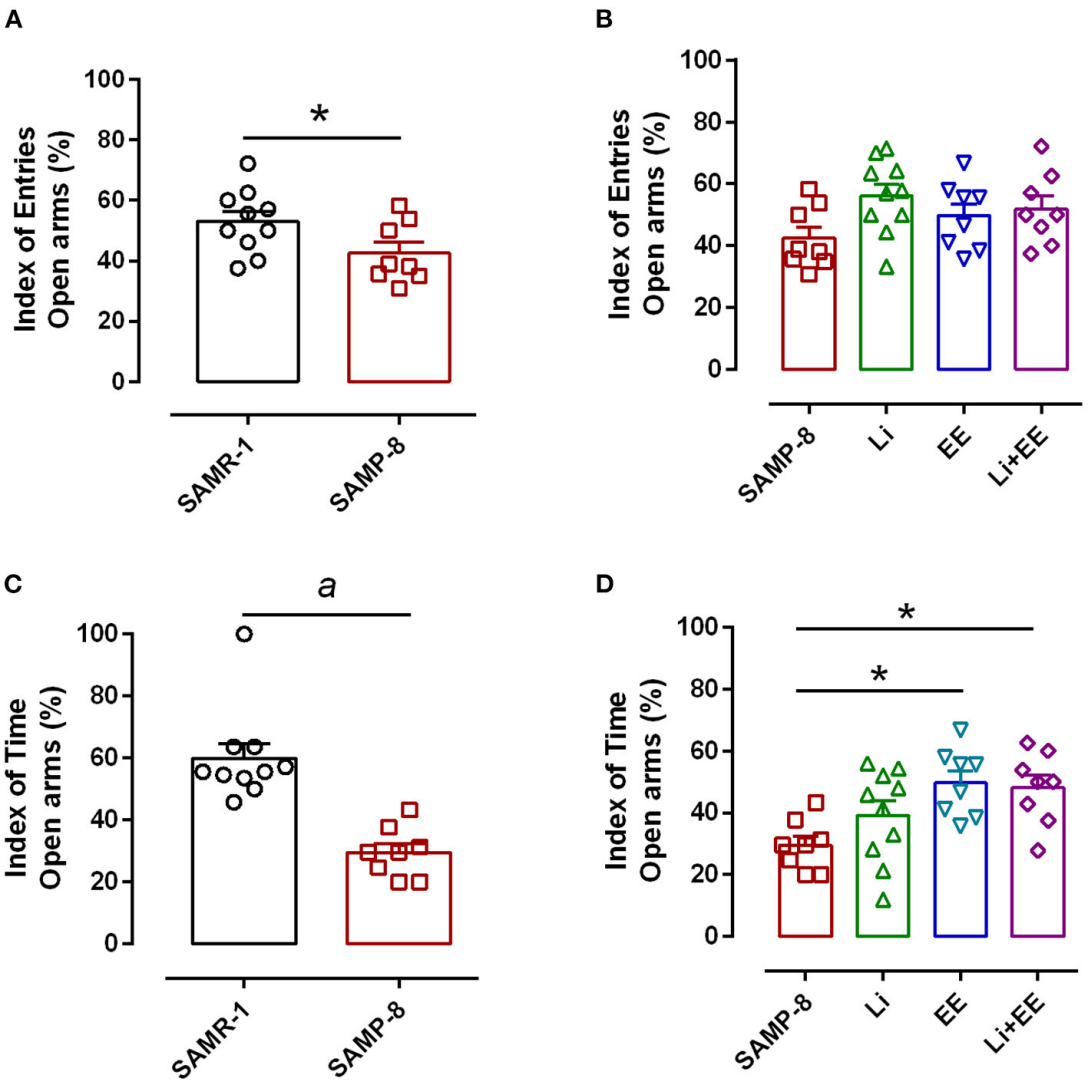
Anxiety-like behavior of Senescence Accelerated Mouse-Prone 8 (SAMP-8) was improved with different strategies**. (A)** Proportion of entries in the open and closed arms between the SAMR-1 and SAMP-8 groups. **(B)** Comparison of the behavior of the treated and untreated SAMP-8 groups with respect to the number of entries in different arms. **(C)** Comparison of the behavior of the SAMR-1 and SAMP-8 groups with respect to the relationship between times spent in the open and closed arms. **(D)** Comparison of the behavior of the treated and untreated SAMP-8 groups with respect to the time spent in different arms. Histograms and vertical bars are means ± standard error of the mean (SEM). *p < 0.05; ^*a*^*p* < 0.0001.

Anxiety-like behavior was evaluated using the elevated plus-maze, comparing the number of entries and time spent in open arms and closed arms. In this respect, a significant reduction of 14.3% in this relationship was observed in the untreated SAMP-8 group when compared with the SAMR-1 group (*t* = 2.148. df = 16, *p* < 0.05, Figure 3A). Among the treated groups, no difference was observed concerning this parameter [*F*_(3,30)_ = 2.257, *p* = 0.1021, Figure 3B]. With respect to the relationship of the time spent in both arms, a decrease of 25.85% was observed in the SAMP-8 group, when compared with the SAMR-1 group (*t* = 5.116, df = 16, *p* < 0.0001, Figure 3C). In this parameter, a significant difference among the SAMP-8 treated groups was observed [*F*_(3,30)_ = 4.887, *p* < 0.01], as the animals from the EE and Li + EE groups spent more time in the open arms when compared with the untreated SAMP-8 group (21.41 and 20.3%, respectively, *p* < 0.05). Animals from the Li group remained about 15% more time in the open arms, but no statistical difference was observed (Figure 3D).

### Different Treatments Are Able to Increase NeuN Expression in SAMP-8

**Figure 4:**
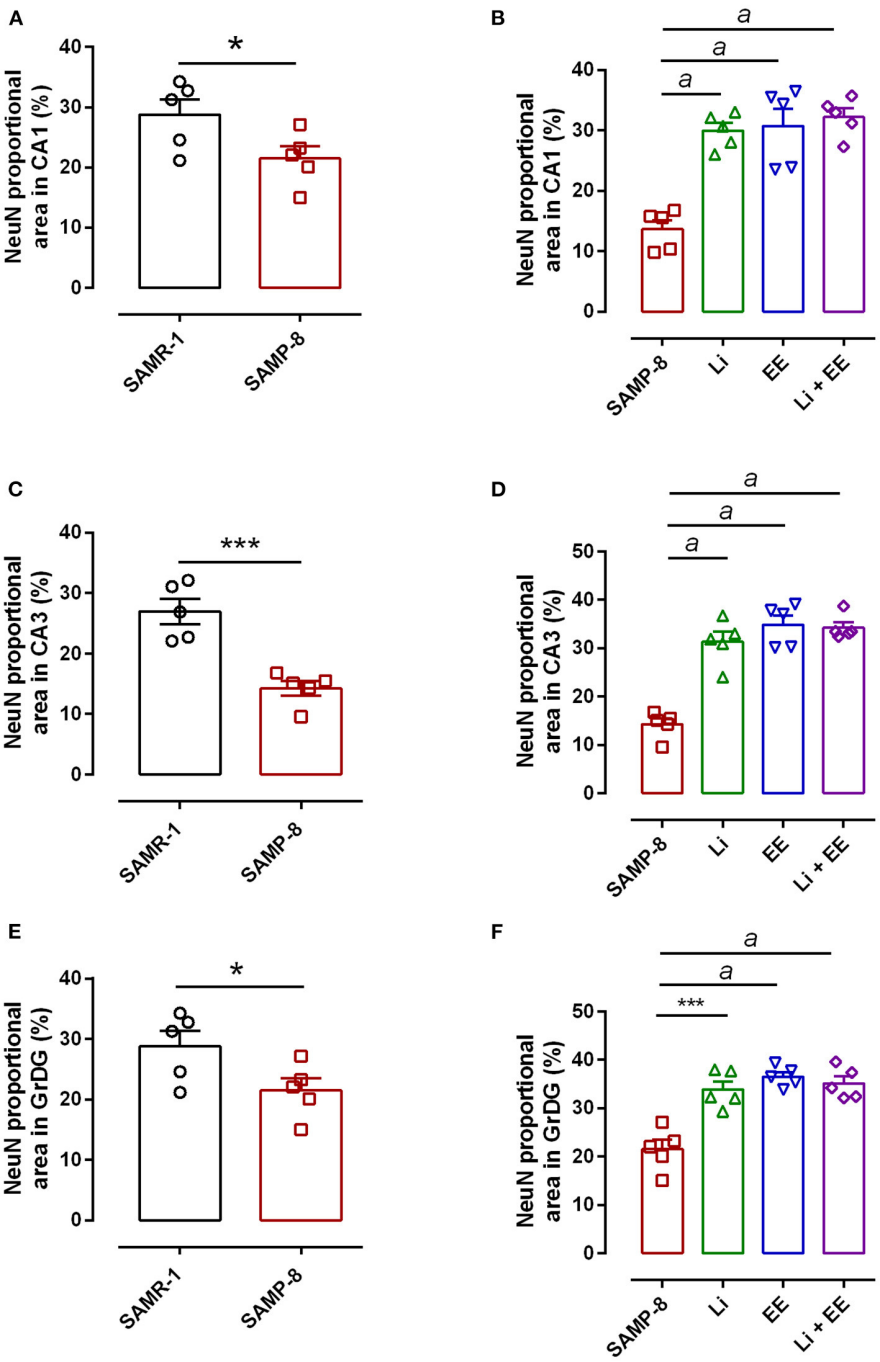
Decrease in the neuronal density of Senescence Accelerated Mouse-Prone 8 (SAMP-8) was avoided with the different strategies. Proportional area of neurons labeled with NeuN in each hippocampal division. **(A,C,E)** comparison between the senescence-accelerated mice resistant (SAMR)-1 and SAMP-8 groups. **(B,D,F)** comparison of treated and untreated SAMP-8 groups. Histograms and vertical bars are means ± standard error of the mean (SEM). *p < 0.05; ***p < 0.001 ^*a*^*p* < 0.0001.

**Figure 5:**
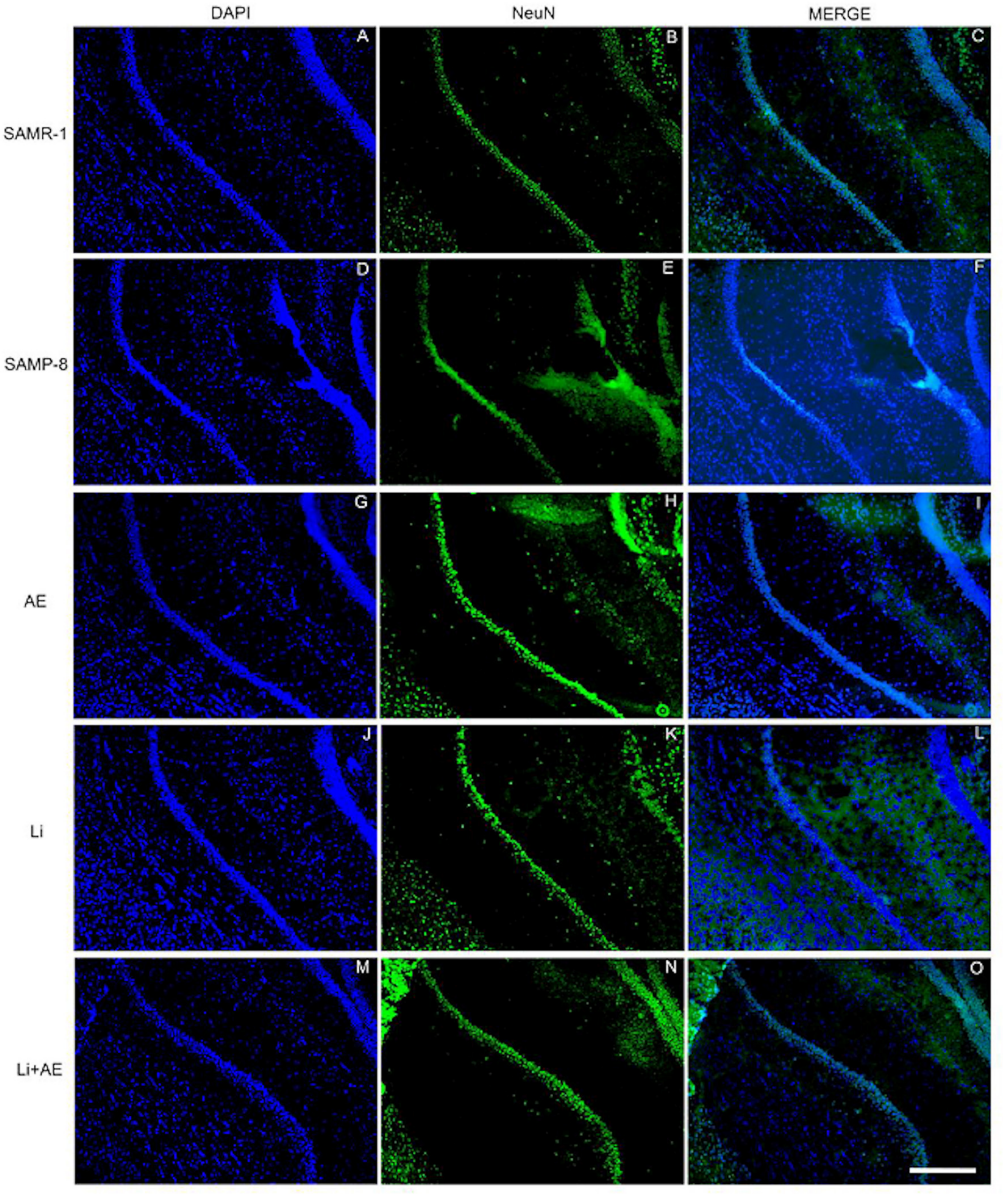
Labeling of NeuN (green) in the CA1 hippocampal region of **(A–C)** senescence-accelerated mice resistant (SAMR)-1, **(D–F)** Senescence Accelerated Mouse-Prone 8 (SAMP-8), **(G–I)** Li, **(J–L)** EE, and **(M–O)** Li + EE mice. Cellular nuclei were labeled with DAPI (blue). Images were obtained using an inverted DMi8 microscope (Leica, Wetzlar, Germany) with a 10× objective. Scale bar: 100 μm.

**Figure 6:**
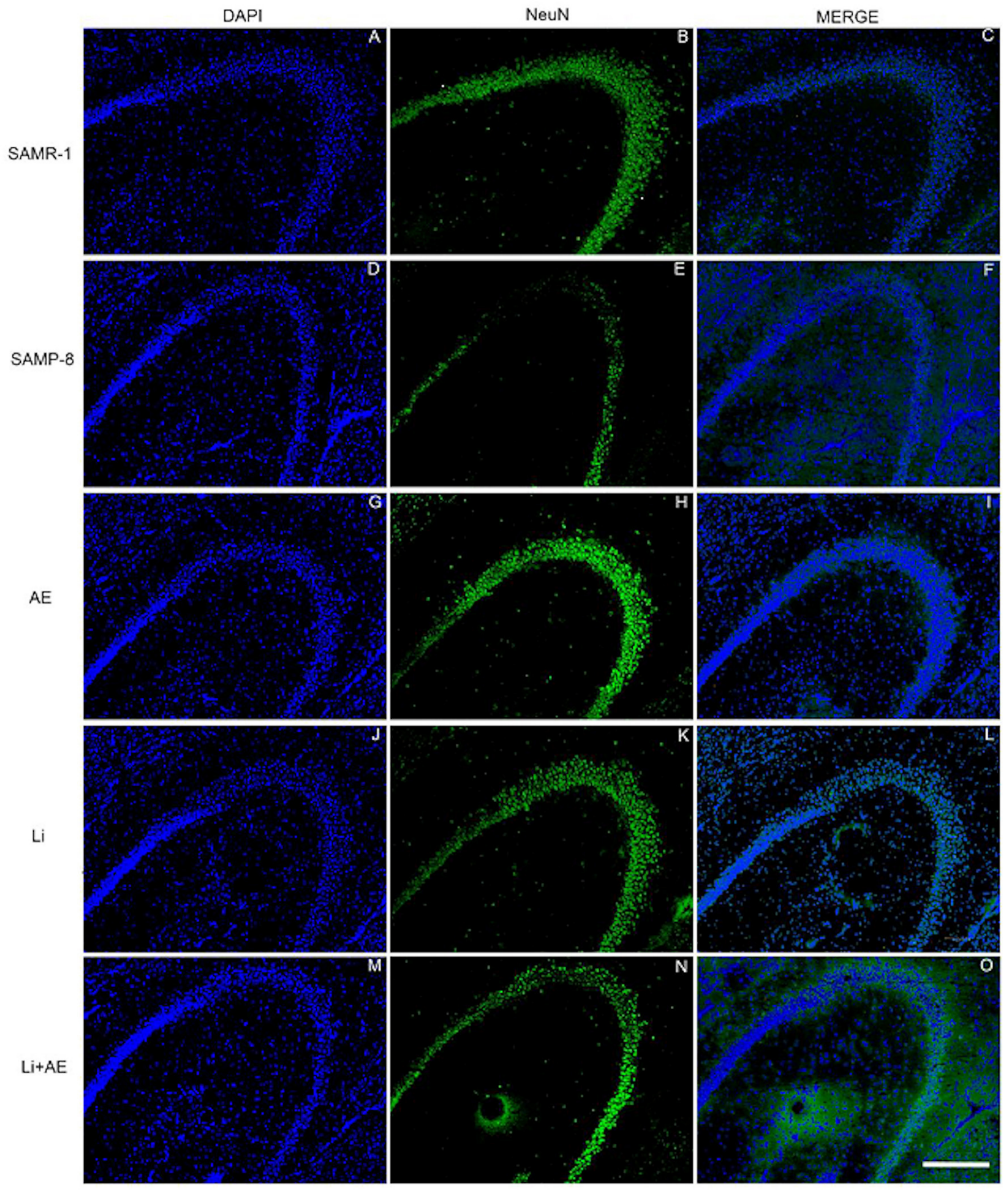
Labeling of NeuN (green) in the CA3 hippocampal region of **(A–C)** senescence-accelerated mice resistant (SAMR)-1, **(D–F)** Senescence Accelerated Mouse-Prone 8 (SAMP-8), **(G–I)** Li, **(J–L)** EE, and **(M–O)** Li + EE mice. Cellular nuclei were labeled with DAPI (blue). Images were obtained using an inverted DMi8 microscope (Leica, Wetzlar, Germany) with a 10× objective. Scale bar: 100 μm.

In the CA1 region, a decrease of 15.7% in the NeuN density in the SAMP-8 group was observed compared with the SAMR-1 group (*t* = 2.264, df = 8, *p* = 0.05, Figure 4A). The SAMP-8 animals submitted to all the treatments presented a significantly higher neuronal density as expressed by interaction among the different treatments [*F*_(1,16)_ = 15.36, *p* < 0.01) (Li: 2.19-fold; EE: 2.25-fold; Li + EE: 2.36-fold, *p* < 0.0001) when compared with the untreated group (Figures 4B, 5). In hippocampal CA3, a significant decrease in the NeuN density of the SAMP-8 group was observed when compared with the SAMR-1 group (11.77%, *t* = 5.282, df = 8, *p* <0.001, Figure 4C). The animals from all the treated groups presented a significantly higher NeuN expression when compared with the animals in the untreated SAMP-8 group, with a significant interaction among the strategies to which the animals were submitted [*F*_(1,16)_ = 28.63, *p* < 0.0001, Li: 2.19-fold; EE: 2.45-fold; Li + AE: 2.4-fold, Figures 4D, 6).

**Figure 7:**
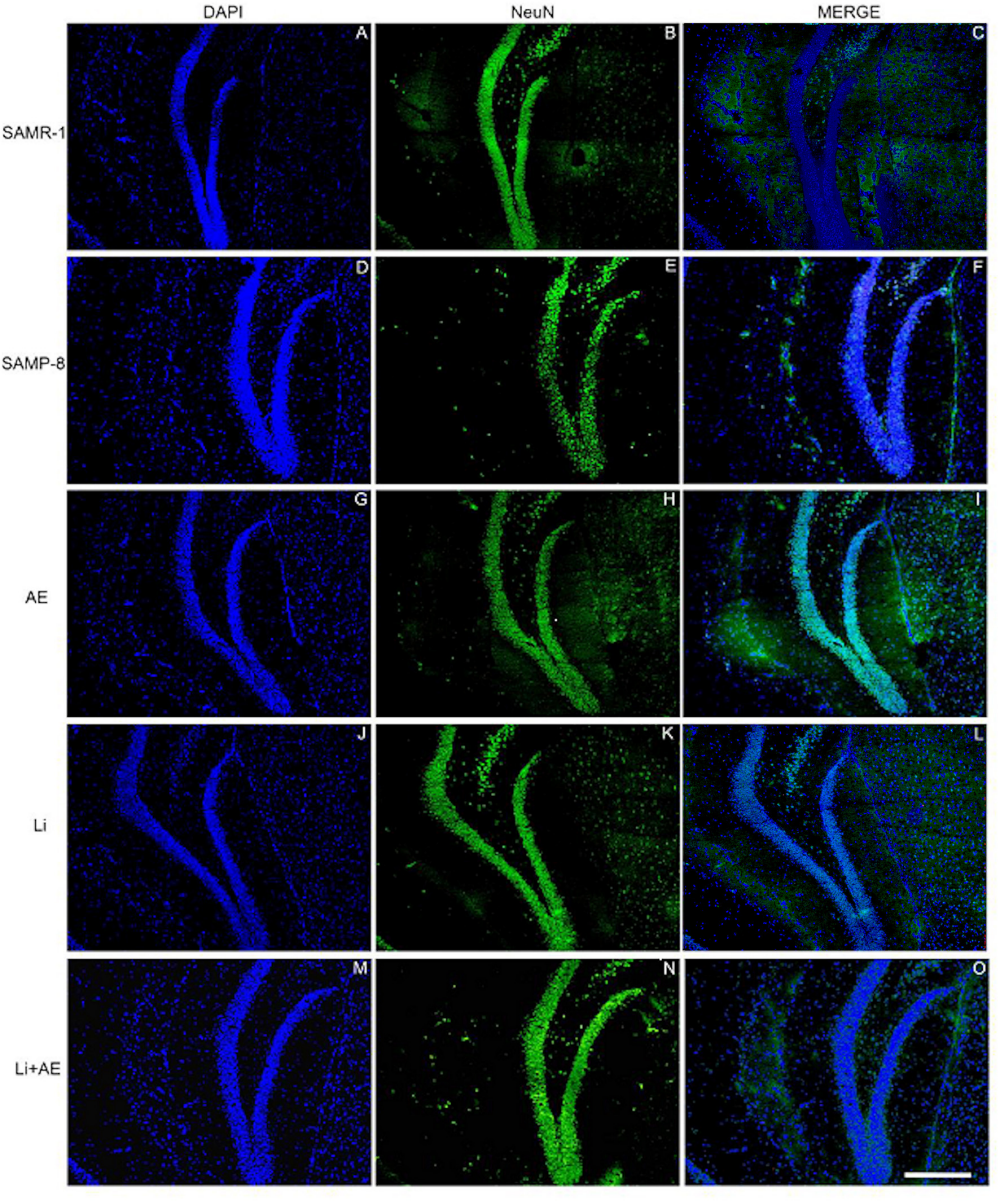
Labeling of NeuN (green) in the GrDG hippocampal region of **(A–C)** senescence-accelerated mice resistant (SAMR)-1, **(D–F)** Senescence Accelerated Mouse-Prone 8 (SAMP-8), **(G–I)** Li, **(J–L)** EE, and **(M–O)** Li + EE. Cellular nuclei were labeled with DAPI (blue). Images were obtained using an inverted Leica DMi8 microscope with a 10× objective. Scale bar: 100 μm.

As observed in other areas, the untreated Senescence Accelerated Mouse-Prone 8 (SAMP-8) animals presented a reduction of 9.22% (Figure 4E, *t* = 2.264, df = 8, *p* = 0.05) in neuronal density when compared with the SAMR-1 group in the GrDG region. With the proposed strategies, no decrease in the NeuN expression in this region was observed, as the density of NeuN labeling was 1.57-fold higher with Li treatment (*p* < 0.001), 1.7 higher in the EE group (*p* < 0.0001), and 1.63 higher in the Li + EE group (*p* < 0.0001), when compared with that of the untreated SAMP-8 group (Figures 4F, 7). The interaction among the strategies used was extremely significant to maintain NeuN density in the SAMP-8 groups [*F*_(1,16)_ = 19.45, *p* < 0.001].

In all the hippocampal areas, no difference was observed in NeuN expression among the treated groups.

### Both the Lithium and the Enriched Environment Were Extremely Effective in Decreasing the Density of the Senile Plaques in SAMP-8 Animals

**Figure 8:**
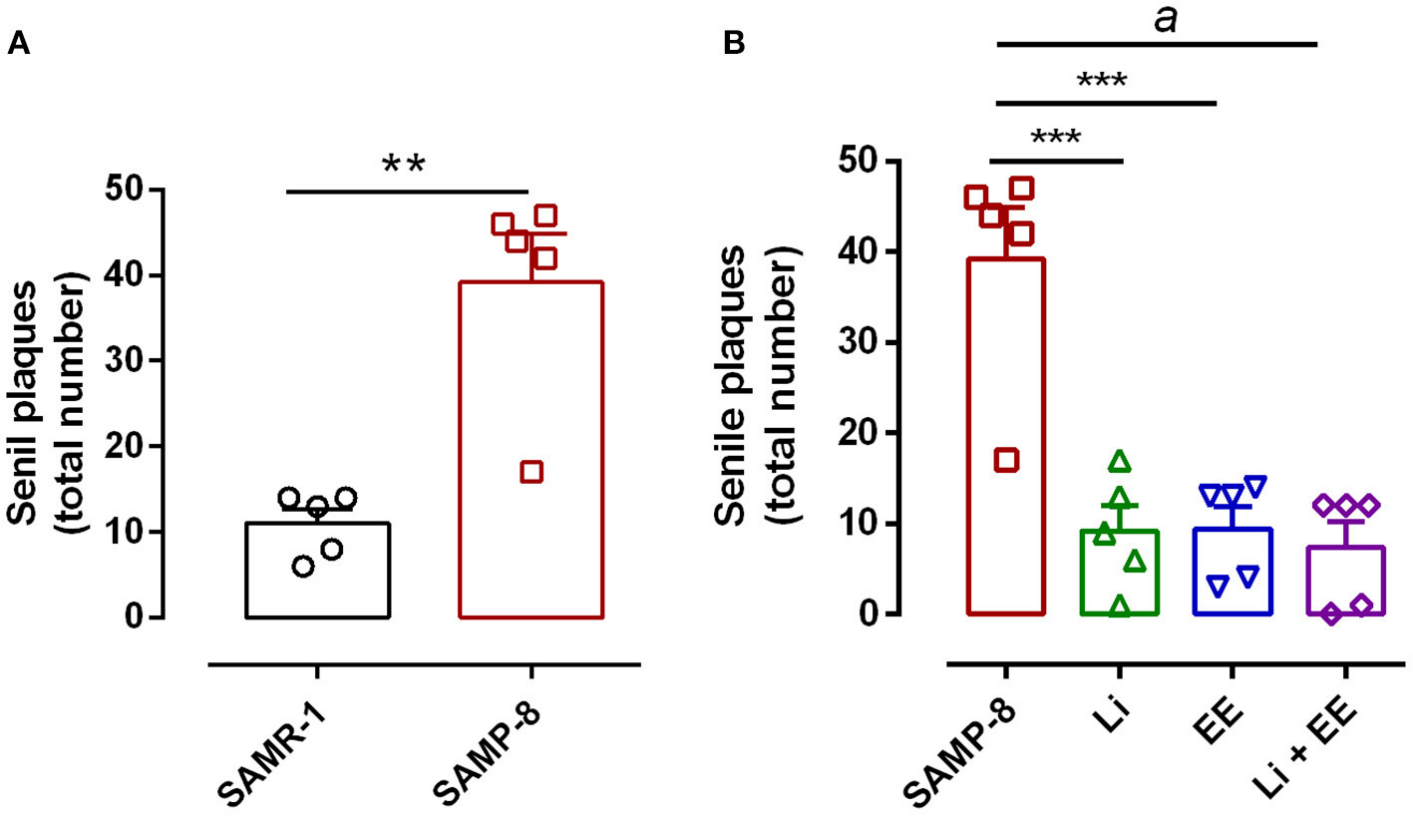
All strategies were able to prevent increased accumulation of senile plaques in the hippocampus of Senescence Accelerated Mouse-Prone 8 (SAMP-8). **(A)** Comparison between senescence-accelerated mice resistant (SAMR)-1 and SAMP-8. **(B)** Comparison of untreated SAMP-8 and treated with different strategies. Histograms and vertical bars are means ± standard error of the mean (SEM). **p < 0.01; ***p < 0.001; ^*a*^*p* < 0.0001.

**Figure 9:**
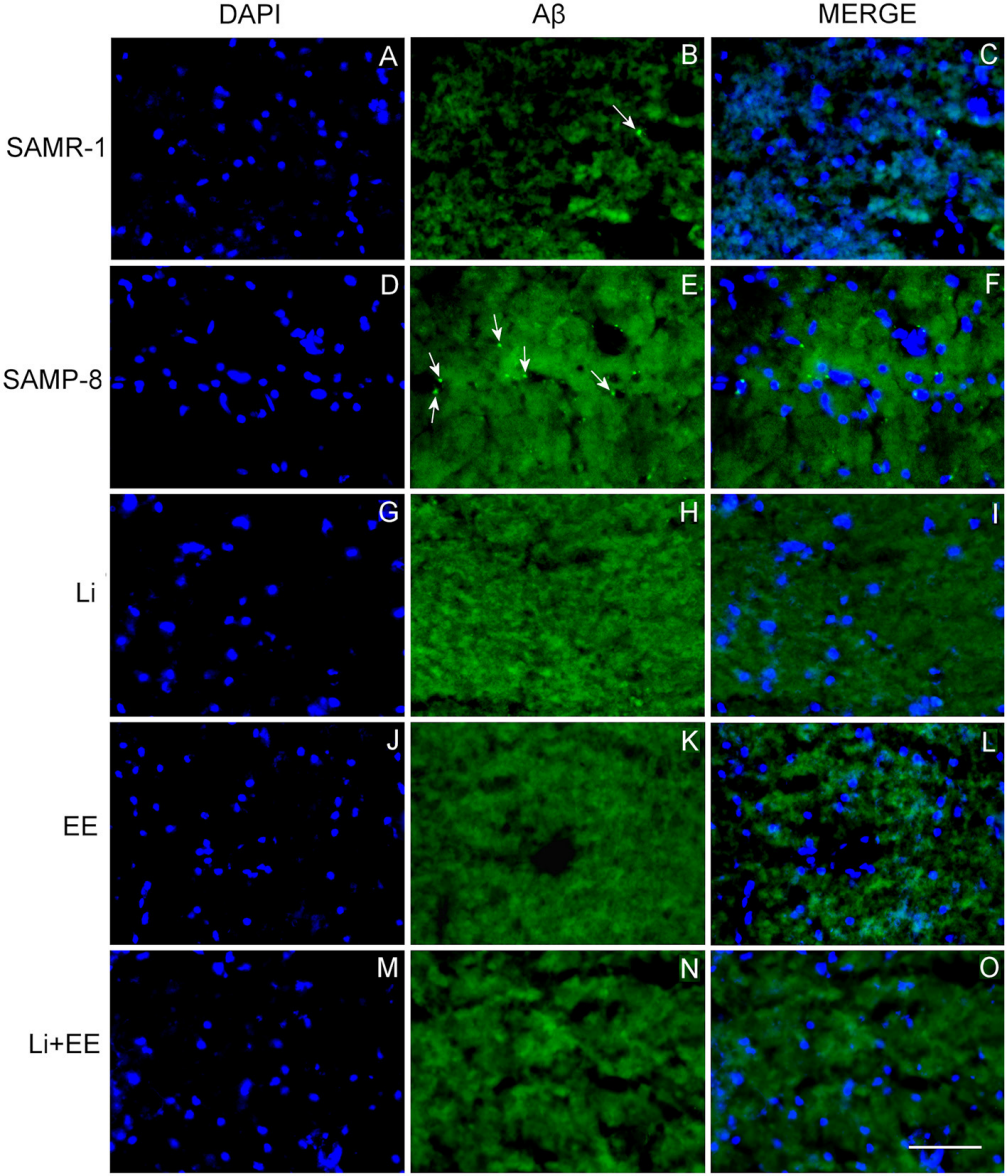
Senile plaques labeled with Thioflavin-S.1% (green) in the hippocampus of **(A–C)** senescence-accelerated mice resistant (SAMR)-1, **(D–F)** Senescence Accelerated Mouse-Prone 8 (SAMP-8), **(G–I)** Li, **(J–L)** EE, and **(M–O)** Li + EE. Cellular nuclei were labeled with DAPI (blue). Images were obtained using an inverted DMi8 microscope (Leica, Wetzlar, Germany) with a 20× objective. Scale bar: 100 μm.

The untreated Senescence Accelerated Mouse-Prone 8 (SAMP-8) animals presented a significant increase of 3.56-fold in the density of senile plaques when compared with the senescence-accelerated mice resistant (SAMR)-1 samples (*t* = 4.812, df = 8, *p* < 0.01) (Figures 8A, 9). All the treatment strategies were efficient, significantly reducing the presence of senile plaques in the SAMP-8 mice when applied along with aging [*F*_(3,16)_ = 17.65, *p* < 0.0001. The following reduction in the density of senile plaques was observed, when compared with the untreated SAMP-8 group: Li: 70.45%, *p* < 0.001; EE: 72.72%, *p* < 0.001; Li + EE: 79.45%, *p* < 0.0001 (Figures 8B, 9). Among the treatments, however, no difference was observed.

### The Different Strategies Used Decreased Neurodegeneration in SAMP-8 Animals

**Figure 10:**
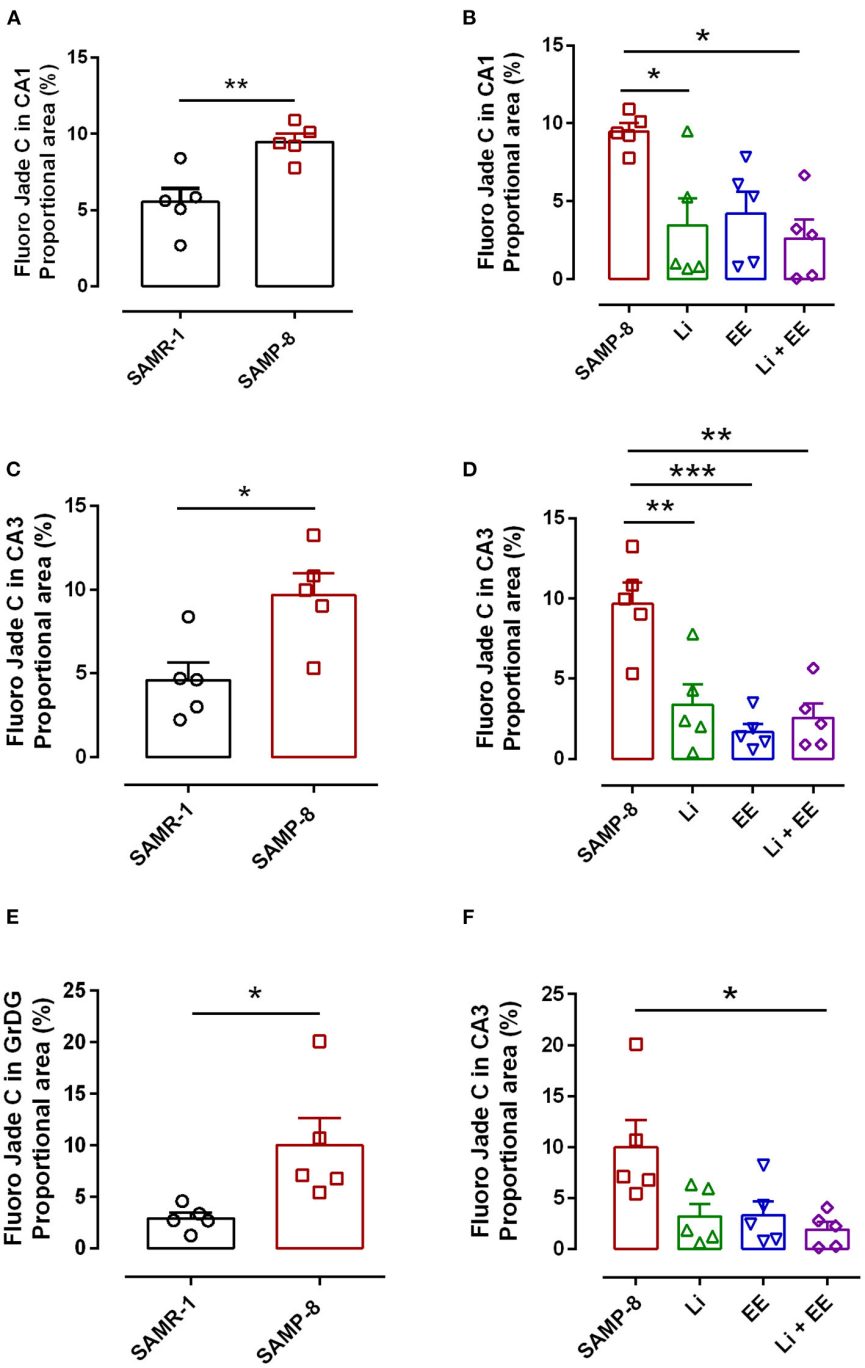
Senescence Accelerated Mouse-Prone 8 (SAMP-8) submitted to the different strategies presented less neurodegeneration in different hippocampal areas. **(A,C,E)** Comparison between senescence-accelerated mice resistant (SAMR)-1 and SAMP-8. **(B,D,F)** Comparison of untreated SAMP-8 and SAMP-8 treated with different strategies. Histograms and vertical bars are means ± standard error of the mean (SEM). *p < 0.05; **p < 0.01; ***p < 0.001.

As a general observation, the Senescence Accelerated Mouse-Prone 8 (SAMP-8) group presented more labeling for the neurodegeneration marker fluorojade than SAMP-8 in all the hippocampal areas studied (CA1: *t* = 3.763, df = 8, *p* < 0.001; CA3: *t* = 3.042, df = 8, *p* < 0.05; GrDG: *t* = 2.607, df = 8, *p* < 0.05, Figures 10A,C,E, respectively).

**Figure 11:**
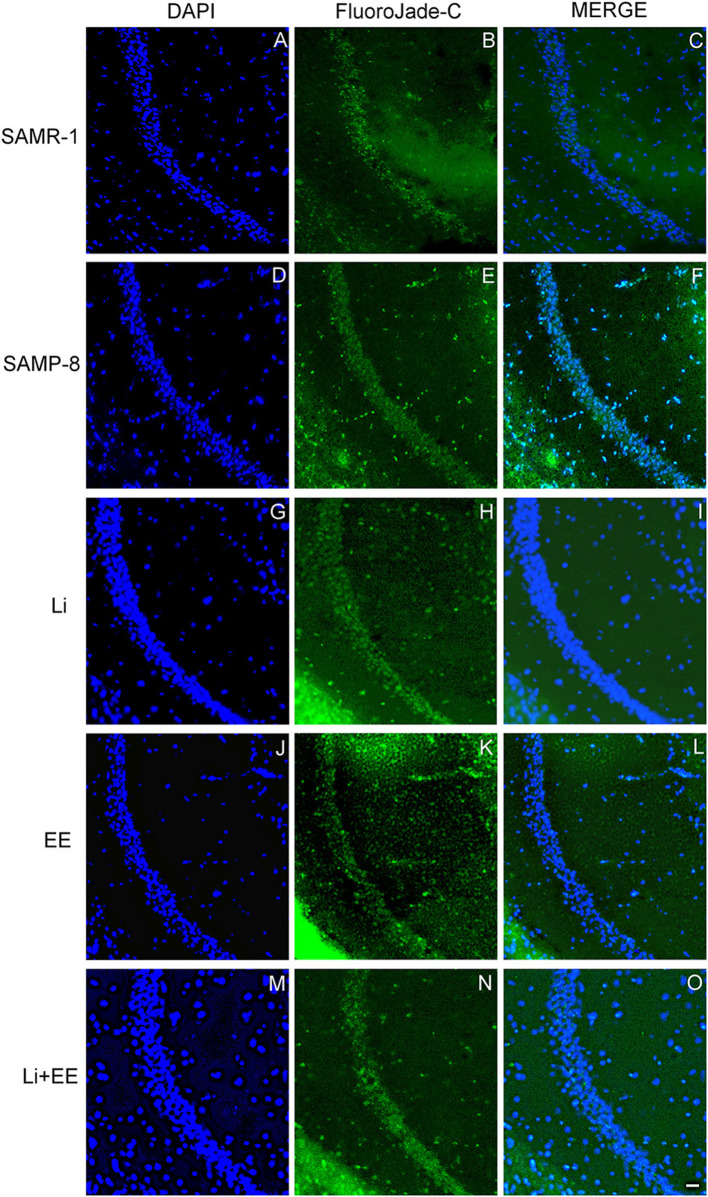
Cells labeled with fluorojade C (green) in the CA1 hippocampal region of **(A–C)** senescence-accelerated mice resistant (SAMR)-1, **(D–F)** Senescence Accelerated Mouse-Prone 8 (SAMP-8), **(G–I)** Li, **(J–L)** EE, and **(M–O)** Li + EE. Cellular nuclei were labeled with DAPI (blue). Images were obtained using an inverted DMi8 (Leica, Wetzlar, Germany) microscope with a 10× objective. Scale bar: 100 μm.

**Figure 12:**
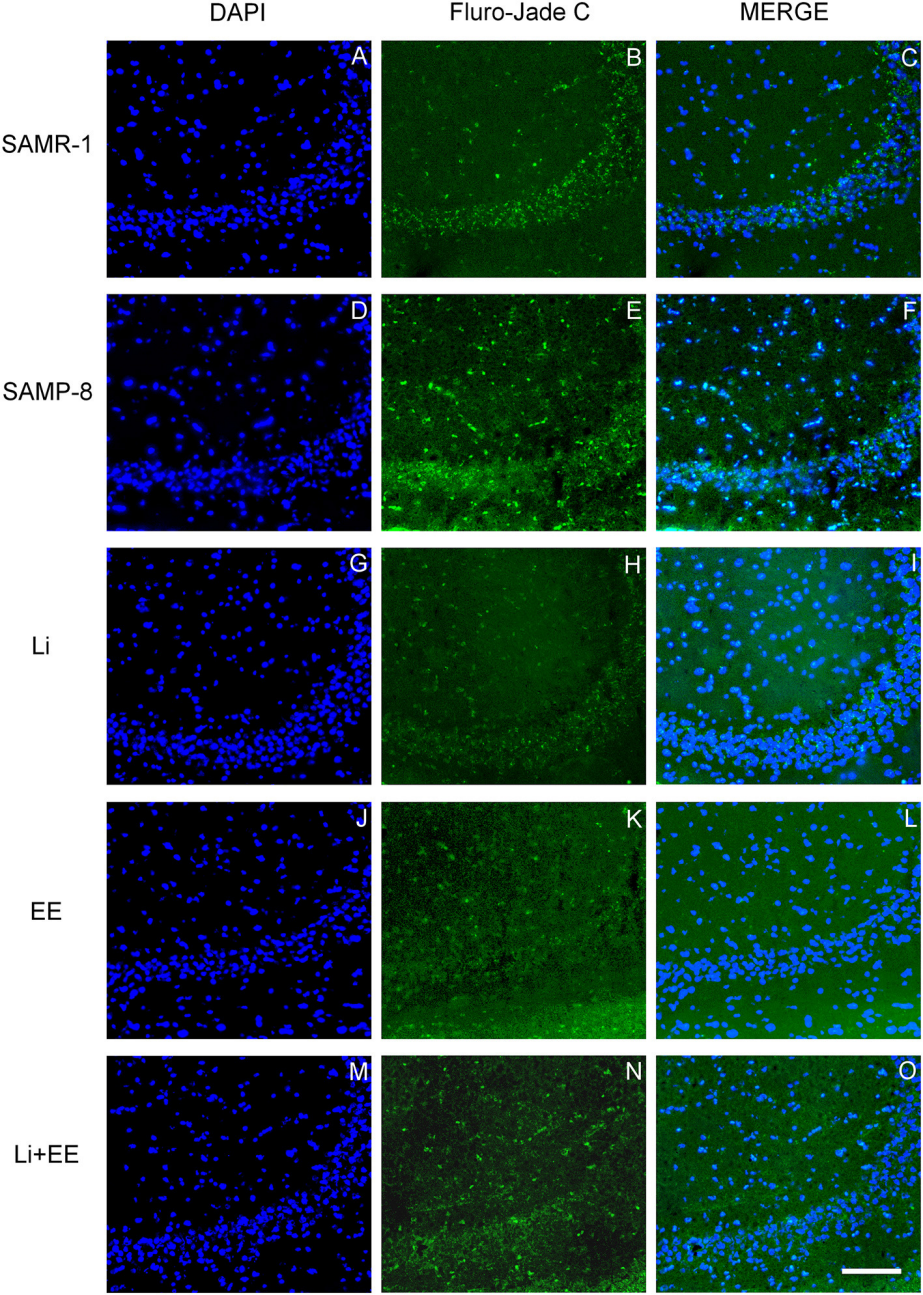
Cells labeled with fluorojade C (green) in the CA3 hippocampal region of **(A–C)** senescence-accelerated mice resistant (SAMR)-1, **(D–F)** Senescence Accelerated Mouse-Prone 8 (SAMP-8), **(G–I)** Li, **(J–L)** EE, and **(M–O)** Li + EE. Cellular nuclei were labeled with DAPI (blue). Images were obtained using an inverted DMi8 (Leica, Wetzlar, Germany) microscope with a 10× objective. Scale bar: 100 μm.

**Figure 13:**
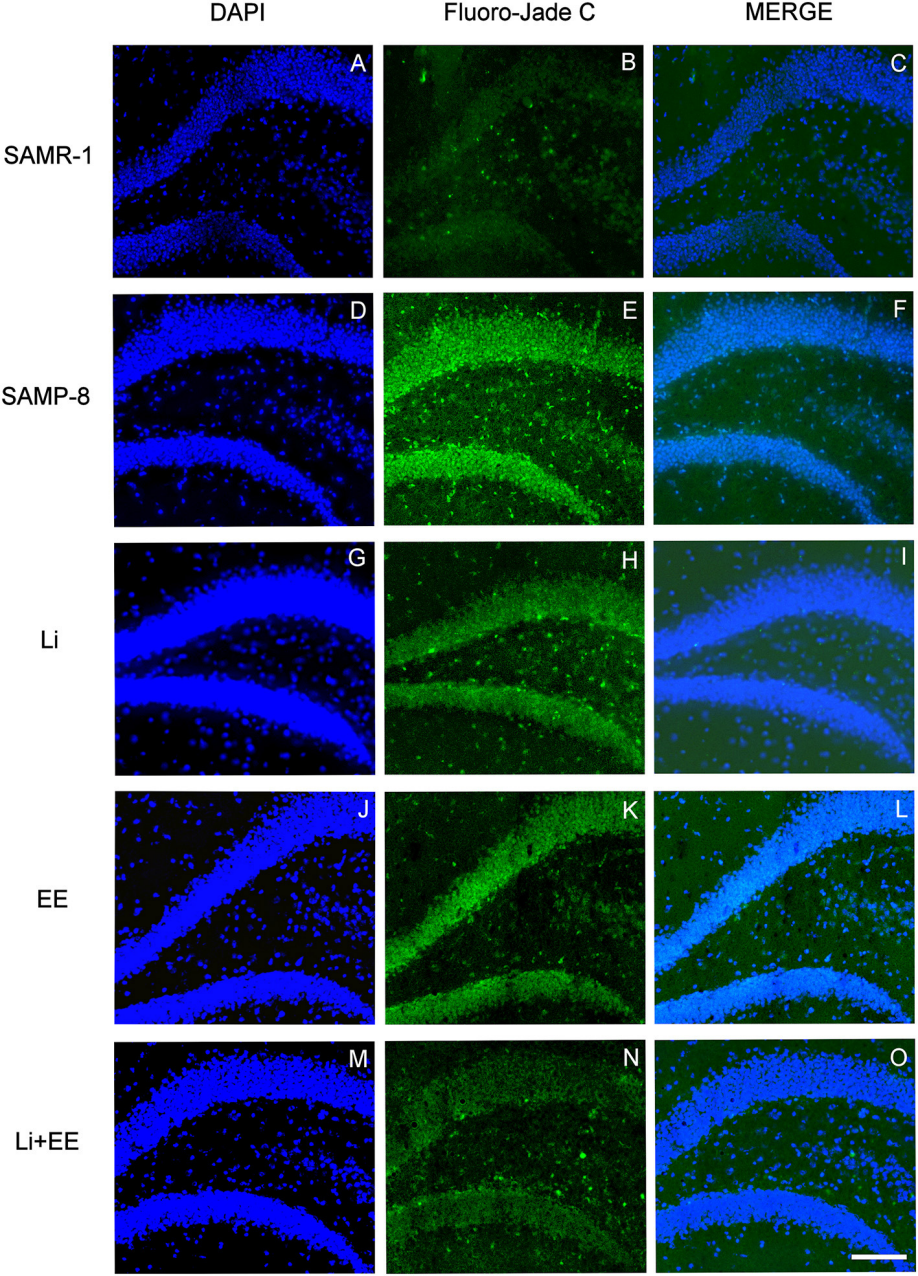
Cells labeled with fluorojade C (green) in the GrDG hippocampal region of **(A–C)** senescence-accelerated mice resistant (SAMR)-1, **(D–F)** Senescence Accelerated Mouse-Prone 8 (SAMP-8), **(G–I)** Li, **(J–L)** EE, and **(M–O)** Li + EE. Cellular nuclei were labeled with DAPI (blue). Images were obtained using an inverted DMi8 (Leica, Wetzlar, Germany) microscope with a 10× objective. Scale bar: 100 μm.

In the CA1 hippocampal area, a significant increase of 1.71-fold (*p* < 0.01) in neurodegeneration was observed in the Senescence Accelerated Mouse-Prone 8 (SAMP-8) group (Figures 10A, 11), whereas the Li and Li + EE treatments promoted a statistically significant reduction in neurodegeneration of 63.56 and 73.45%, respectively (*p* < 0.05). EE alone also promoted a reduction in neurodegeneration, but with no statistical significance (Figures 10B, 11). In the CA3 area, the SAMP-8 samples presented a 2.11-fold greater increase in neurodegeneration than the SAMR-1 samples (*p* < 0.05, Figures 10C, 12). In this region, all the treatments promoted a significant reduction in neurodegeneration when compared with untreated SAMP-8 (Li: 92.42%, *p* < 0.01; EE: 91.42%, *p* < 0.001; Li + EE: 92.21%, *p* < 0.01, Figures 10D, 12). Similarly, SAMP-8 presented a significantly greater area under degenerative process in the GrDG region (3.43-fold, *p* < 0.05) when compared with SAMR-1 (Figures 10E, 13). All the treatments promoted a reduction in neurodegeneration; however, only the Li + EE group presented a significant reduction of 81.04% (*p* < 0.05) when compared with untreated SAMP-8 (Figures 10F, 13). No difference among the groups was observed.

### The Different Treatments Reversed the Expression of CAMK IV in SAMP-8

**Figure 14:**
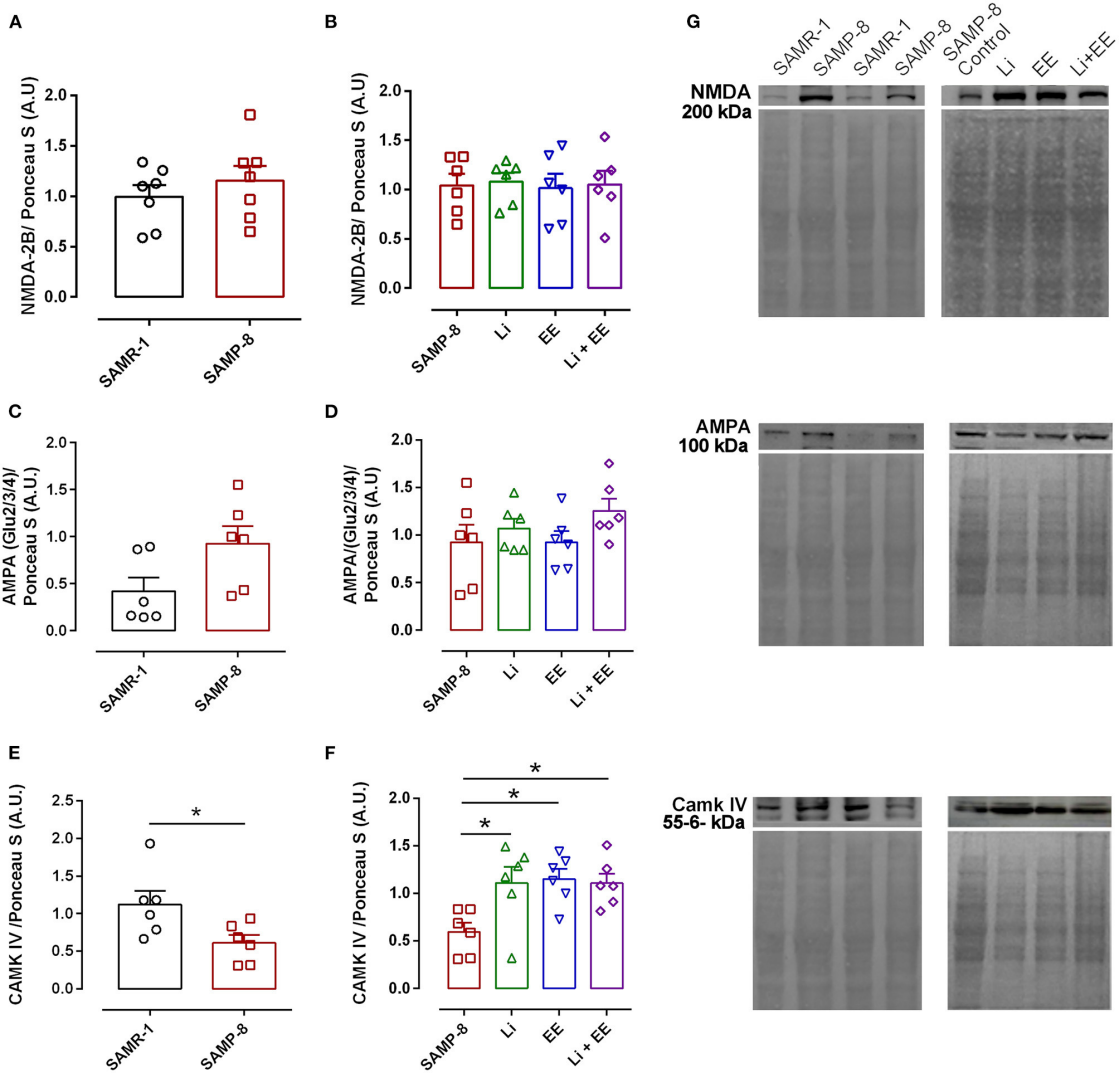
Different strategies increased the density of CAMK IV measured in hippocampal tissue. The density of proteins NMDA-2B, AMPA, and CAMK IV in the hippocampal samples of SAMR-1 compared with SAMP-8 **(A,C,E,G)** and untreated Senescence Accelerated Mouse-Prone 8 (SAMP-8) and SAMP-8 treated with microdosing lithium, submitted to microdosing lithium, enriched environment, or combined treatment **(B,D,F,G)**. *p < 0.05.

The analysis of NMDA-2B receptor density revealed no difference between the senescence-accelerated mice resistant (SAMR)-1 and Senescence Accelerated Mouse-Prone 8 (SAMP-8) samples (*t* = 0.8415, df = 12, *p* = 0.4165, Figure 14A). In the same way, the treatments did not influence the density of this receptor in the brain samples of the treated SAMP-8 animals [*F*_(3,20)_ = 0.04647, *p* = 0.9863, Figure 14B]. Nevertheless, the SAMP-8 group presented an increase of 2.2-fold (*t* = 2.126, df = 10, *p* = 0.0596) in the density of the AMPA receptor, when compared with the SAMR-1 group, but the treatments did not affect this parameter (Figures 14C,D). With respect to the density of the CAMK IV protein, a significant reduction of 54.5% (*t* = 2.418, df = 10, *p* < 0.05) was observed in the SAMP-8 samples when compared with the SAMR-1 samples. The treated animals showed maintenance of density, as a higher density of this protein was observed in all the groups when compared with the untreated animals [Li: 1.86-fold; EE: 1.93-fold; Li + AE: 1.86-fold, *F*_(3,20)_ = 4.628, *p* < 0.05, Figure 14]. No difference among the groups was observed (Figure 14).

### The Combination of the Enriched Environment Plus Lithium Increased the Number of Synapses in SAMP-8 Mice

**Figure 15:**
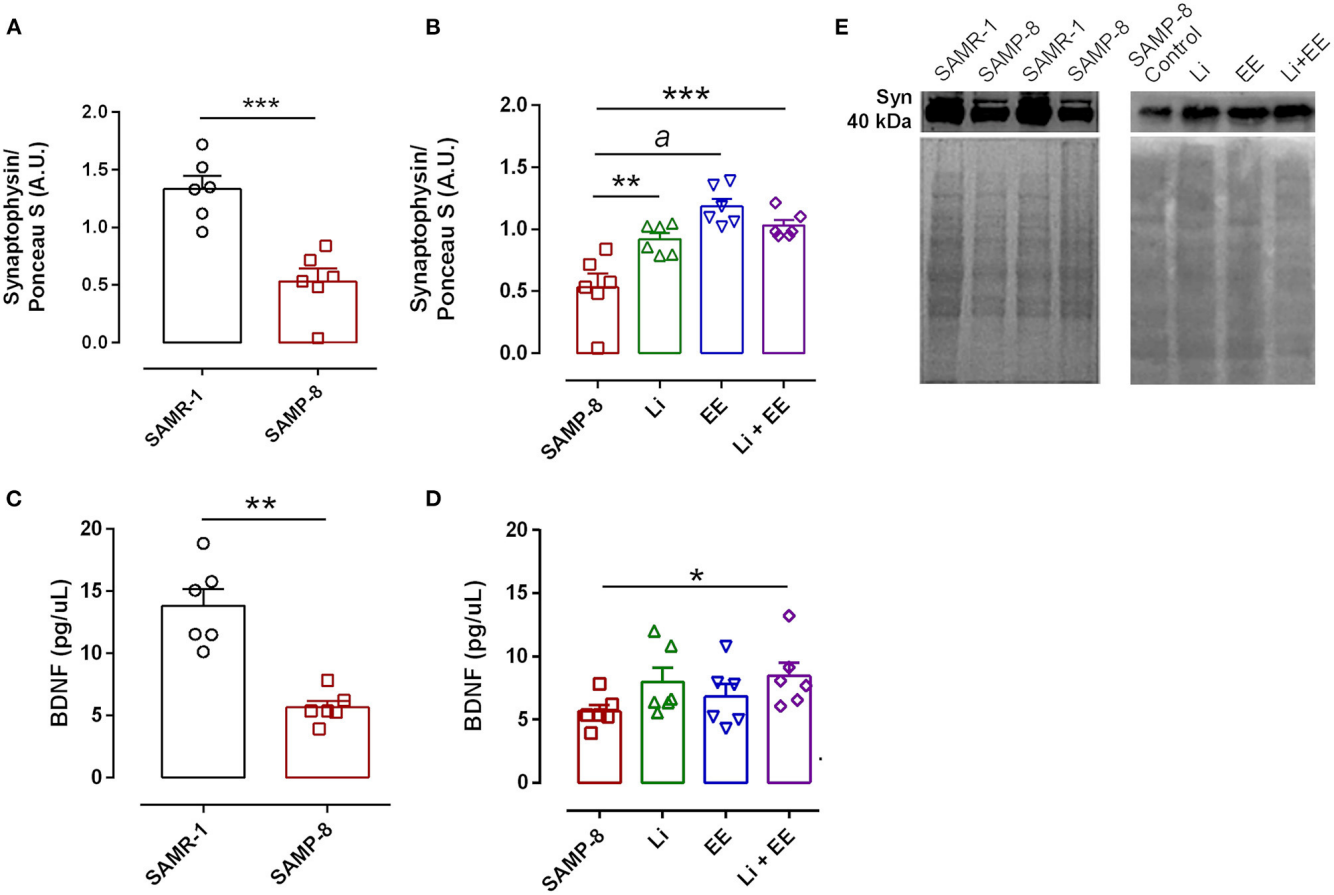
Synaptophysin density, but not brain-derived neurotrophic factor (BDNF), was increased in Senescence Accelerated Mouse-Prone 8 (SAMP-8) submitted to the different strategies. Density of synaptophysin **(A,B,E)** measured with Western-blot assays) and BDNF **(C,D)** measured with enzyme-linked immunosorbent assays (ELISAs) in the samples of SAMR-1, untreated SAMP-8, and SAMP-8 from the Li, EE, and Li + EE groups. *p < 0.05; **p < 0.01; ***p < 0.001; ^*a*^*p* < 0.0001.

In the analysis of the density of synaptic terminals, the SAMP-8 group presented a significantly lower synaptophysin density when compared with the senescence-accelerated mice resistant (SAMR)-1 group (58.68%, *t* = 5.101, df = 10, *p* < 0.001, Figures 15A,E). The treatments maintained the density of the synaptic terminals, with significant increases in synaptophysin density in the Li (2.24-fold, *p* < 0.01), EE (1.73-fold, *p* < 0.0001), and Li + EE (1.94-fold, *p* < 0.001) groups, when compared with the untreated SAMP-8 group [*F*_(3,20)_ = 14.82, *p* < 0.0001, Figures 15B,E]. No difference among the treated groups was observed. Concerning the density of the neurotrophin BDNF, a significant decrease of 59.15% of this protein was observed in the SAMP-8 samples, when compared with the SAMR-1 group (*t* = 5.627, df = 10, *p* < 0.001, Figure 15C). In the hippocampal samples, no significant difference was observed among the groups [*F*_(3,20)_ = 1.74, *p* = 0.191). However, concerning the EE and Li + EE groups, a significant increase in the density of BDNF was observed when compared with the untreated SAMP-8 group (2.08- and 3.09-fold, respectively, *p* < 0.05, Figure 15D). In the same way, the animals treated with Li also showed an increase in the density of BDNF (1.83-fold), but this was not statistically significant.

## Discussion

This study aimed to evaluate whether the combined use of two strategies to promote healthy aging would lead to better results than a single strategy. To explore this hypothesis, a mouse model of accelerated aging (SAMP-8) was submitted to different strategies (here called “treatments”) during the aging process, and parameters related to memory, anxiety, and long-term potentiation were analyzed. The SAMR-1 group was not submitted to the treatments, as in the previous study we showed that chronic treatment with microdose lithium (for 16 months) promoted neuroprotective effects only in transgenic mice for AD, which presented a neurodegenerative process. Wild animals did not show any improvement in memory or the biomarkers for neuroprotection (Nunes et al., 2015). Also, in another study performed by the research team (data not published), chronic treatment of C57Bl/6 mice with the same lithium dose, for 10 months, did not change the markers of neuroprotection as well. In this way, we opted to compare SAMP-8 with SAMR-1 to characterize the decrease in memory and the anxiety-like behavior of SAMP-8 and to show the alteration in cell density, neurodegeneration, and presence of senile plaques. As observed in the data generated in this study, chronic treatment of SAMP-8 significantly changed their anxiety-like behavior and prevented brain degeneration observed in untreated mice. In a general analysis, it is possible to describe that the SAMP-8 group presented behavioral problems and alteration of neurotransmission when compared with their control, SAMR-1. In the analysis of spatial memory, the Li + EE group showed a significant increase in the time spent in the right quadrant (the location of the escape hole) in the Barnes maze test at 8 months of age than the animals submitted to only one of these treatments, showing that the beneficial effects previously observed with these strategies (Baraldi et al., 2013; Nunes et al., 2015; Balthazar et al., 2018) can be improved if they are combined. The absence of significant differences observed at 10 months of age can be explained due to the habituation of the animals to the Barnes' maze, which makes animals reduce their exploration.

One factor that is linked to memory deficits is anxiety (Mah et al., 2015). In this study, it was shown that the untreated SAMP-8 mice showed higher levels of anxiety-like behavior than the SAMR-1 control mice. Similar behavior has been observed previously and may be related to the fact that the SAMP-8 mice show more activity when in contact with new environmental stimuli and show more anxiety than the SAMR-1 mice, probably because of the presence of different colors, forms, and objects that they are not used to Brandewiede et al. (2005). In addition to different environmental factors, increased age in these animals also directly influences increased anxiety, as described in a previous study (Chen et al., 2007). In this study, the EE and Li + EE animals spent significantly more time in the open arms than the untreated animals, indicating that these animals were more prone to exposure to unknown environments and, therefore, were less anxious. The reduction in anxiety after treatment with microdose lithium was observed previously by the research team (Nunes et al., 2015), both in a transgenic animal model of AD and in C57Bl/6 (wild) animals. Similarly, it was found that environmental enrichment promoted a reduction in anxiety in the transgenic mice model for AD (Benaroya-Milshtein et al., 2004; Pietropaolo et al., 2014). In the same way, the combined treatment seems to improve the behavior of the SAMP-8 mice.

Behavioral changes were accompanied by changes in the hippocampus of the animals. Neuronal density was significantly reduced in the hippocampus of the untreated SAMP-8 animals when compared with the SAMR-1 mice, and all the strategies (combined or not) were able to significantly maintain neuronal density. This suggests that even if no significant improvement in spatial memory was observed, adopting a strategy to change the regular lifestyle can protect the brain from neuronal loss. This was also confirmed with respect to the analysis of senile plaque density and the degree of neurodegeneration. The SAMR-1 group presented senile plaque deposition, which was somewhat unexpected. However, some previous studies have shown that at 9 months of age the SAMR-1 mice showed plaque deposition in the hippocampus but with no cognitive deficits (Del Valle et al., 2010). In human beings, although greater deposition of senile plaque is linked to a diagnosis of AD, older adults with no clinical diagnosis of the disease may also show amyloid-β deposition in the brain with no memory deficits (Aizenstein et al., 2008). In this study, there was an increase in senile plaque in the SAMP-8 group in comparison with the SAMR-1 group, and all the treatments reduced the density of plaques in the SAMP-8 mice. This could contribute to better memory performance. It was found that the untreated SAMP-8 mice presented more neurodegeneration and a significant decrease in neuronal density in the GrDG, CA1, and CA3 hippocampal areas when compared with the SAMR-1 control, factors contributing to the behavioral observations. This could also be related to the increase in senile plaque deposition in the hippocampus. The presence of senile plaque, mainly composed of Aβ1-42 peptides, leads to greater neurotoxicity and neurodegeneration (Pike et al., 1993; Kaku et al., 2003; Oakley et al., 2006). Individual treatments with lithium and environmental enrichment promoted increases in neuronal density in GrDG, CA1, and CA3 and decreased neurodegeneration in the same areas when compared with the hippocampal samples of the untreated SAMP-8 mice. These observations are in agreement with the literature, as it has previously been shown that environmental enrichment with 2 month-old SAMP-8 and in a transgenic mouse model for AD promoted a reduction in neuronal loss (Akiguchi et al., 2017; Griñán-Ferré et al., 2018). Lithium also promoted a neuroprotective effect on GrDG, CA1, and CA3, reducing neurodegeneration and preserving neuronal density. C57Bl/6 animals with induced neurodegeneration and a transgenic mouse model of AD also presented increased neuronal density after treatment with lithium (Nunes et al., 2015; Relaño-Ginés et al., 2018). The animals treated with the combined strategies in this study also presented neuroprotection, but this combination was not more effective than the treatments alone.

Long-term memory formation, as observed in this study during the aging process of mice, depends on changes in neuronal structure and connection, known as long-term potentiation (LTP) (Bliss and Collingridge, 1993; Nicoll, 2017). In this biological process, the activation of the *N-*methyl D-aspartate (NMDA) and alfa-amino-3-hydroxy-methyl-5-4-isoxazolpropionic (AMPA) receptors is critical for the establishment of the LTP molecular pathway (Bliss and Collingridge, 1993). Other studies have already shown dysfunction in LTP in SAMP-8 when compared with SAMR-1 (Taniguchi et al., 2015a,b). In this study, although there was a significant reduction in memory behavior in the untreated SAMP-8 mice when compared with the SAMR-1 mice, no difference in the NMDA-2B receptor was observed. One possible explanation for this is that this specific NMDA receptor has particularity in brains with a neurodegenerative process. The expression of NMDA-2B may be reduced in the neuronal synaptic surface and increased in the extra-synaptic region in SAMP-8 because of the high density of senile plaque in these animals (Snyder et al., 2005; Parsons and Raymond, 2014; Bading, 2017). In this study, however, it was not possible to verify the cellular localization of NMDA-2B. The SAMP-8 group also presented a greater density of AMPA receptors when compared with the SAMR-1 group. Similar data were observed in patients with AD, with no change in NMDA receptor density (Marcello et al., 2013), and in a transgenic mouse model for AD (Megill et al., 2015), reinforcing the hypothesis that the increase in the density of these receptors may promote neurodegeneration. In the brain samples of the treated SAMP-8 mice, no difference in the density of AMPA receptors was found. With respect to the LTP cellular pathway, the untreated SAMP-8 mice presented a lower density of the intracellular protein CAMK IV, when compared with the SAMR-1 control. A decrease in this protein suggests a reduction in early LTP (Tomobe and Nomura, 2009), which could explain the worse performance of these animals in the Barnes maze. However, the treatments applied to the SAMP-8 mice promoted an increase in the density of CAMK IV when compared with the untreated animals, although no significant changes in memory behavior were observed in the Barnes maze. As observed previously with transgenic mice for AD, environmental enrichment promoted an increase in CAMK IV, with an increase in pCREB (Hu et al., 2013). The same was observed in lymphoblast cells treated with lithium, which presented an increased density of pCREB (Alda et al., 2013). Combined treatment was not more effective than treatments alone but showed efficacy, as it promoted an increase in CAMK IV density. With respect to LTP and its structural consequences, a reduction in the density of synaptophysin was observed in SAMP-8 and has been related to a low capacity for learning and memorizing, and has been demonstrated in other animal species (Wang et al., 2007). Both treatments applied during the aging process maintained the synaptic density of the SAMP-8 mice, as was observed previously with the lithium treatment of naïve rats after traumatic brain injury (Carlson et al., 2017) and with the exposure of rats to an enriched environment (Saito et al., 1994). In the untreated SAMP-8 samples, a significant reduction in the BDNF density of the hippocampus was observed when compared with the hippocampus of the SAMR-1 mice, as was observed previously (Chan et al., 2017). In this study, when the animals were submitted to the different strategies, all the groups showed increases in BDNF density, and a significant increase was observed when the animals were submitted to the combined strategies. Earlier, the group of the authors has shown that transgenic mice for AD also presented increased density of BDNF after treatment with microdose lithium (Nunes et al., 2015) or exposure to an enriched environment (Baraldi et al., 2013). Also, an increase in BDNF serum levels was already reported after treatment of patients with AD with lithium (Leyhe et al., 2009).

## Conclusion

The group of the authors has previously shown that treatment with microdoses of lithium and environmental enrichment are effective strategies to prevent the evolution of Alzheimer's disease in different animal models. In this study, we show that the combined use of these strategies is effective in promoting memory retention and reducing anxiety-like behavior, although we did not see an increase in the potency of neuroprotection, as all the treatments maintained the number of neurons, alongside a reduction in senile plaques density and neurodegeneration in the SAMP-8 treated groups. The treatments also contributed to maintaining long-term potentiation and, consequently, long-term memory, with the combined treatment being as or more effective than the treatments applied alone. This conclusion is extremely valuable, as in everyday life individuals may be exposed to multiple positive stimuli (nutritional, intellectual, sensorial, emotional, physical, etc.), but this is random rather than through a plan. Moreover, this study highlights the fact that those who lack a variety of stimulation during their lives, and particularly individuals with a genetic tendency to develop memory and emotional alterations, are at increased risk. In this way, it is suggested that individuals should be more conscious of what these strategies are and apply them more systematically. In other words, changing the lifestyle to increase environmental enrichment together or not with microdose lithium treatment, should be strongly considered as a plan for a better life, for health span. We hope that the results of this study can help to inform future programs devoted to healthy aging.

## Data Availability Statement

The original contributions presented in the study are included in the article/Supplementary Material, further inquiries can be directed to the corresponding author/s.

## Ethics Statement

The animal study was reviewed and approved by Animal Ethics Committee of the Santa Casa de Sáo Paulo School of Medical Sciences (protocol 012/2016).

## Author Contributions

HM: conceptualization, methodology, validation, formal analysis, investigation, writing—original draft, and visualization. AP and MP: investigation and formal analysis. GA: investigation, methodology, and formal analysis. MT: investigation, formal analysis, writing—review and editing, and supervision. EA: methodology and writing—review and editing. HB: methodology, validation, resources, writing—review and editing, and funding acquisition. TV: conceptualization, methodology, validation, formal analysis, resources, writing—original draft, writing—review and editing, supervision, project administration, and funding acquisition. All authors contributed to the article and approved the submitted version.

## Conflict of Interest

The authors declare that the research was conducted in the absence of any commercial or financial relationships that could be construed as a potential conflict of interest.

## Publisher's Note

All claims expressed in this article are solely those of the authors and do not necessarily represent those of their affiliated organizations, or those of the publisher, the editors and the reviewers. Any product that may be evaluated in this article, or claim that may be made by its manufacturer, is not guaranteed or endorsed by the publisher.
