# Supplementary figures and images for: Combined Neuroprotective Strategies Blocked Neurodegeneration and Improved Brain Function in Senescence-Accelerated Mice
Source: Front Aging Neurosci. 2021 Aug 23;13:681498. doi: 10.3389/fnagi.2021.681498 (PMC8419356; doi:10.3389/fnagi.2021.681498)

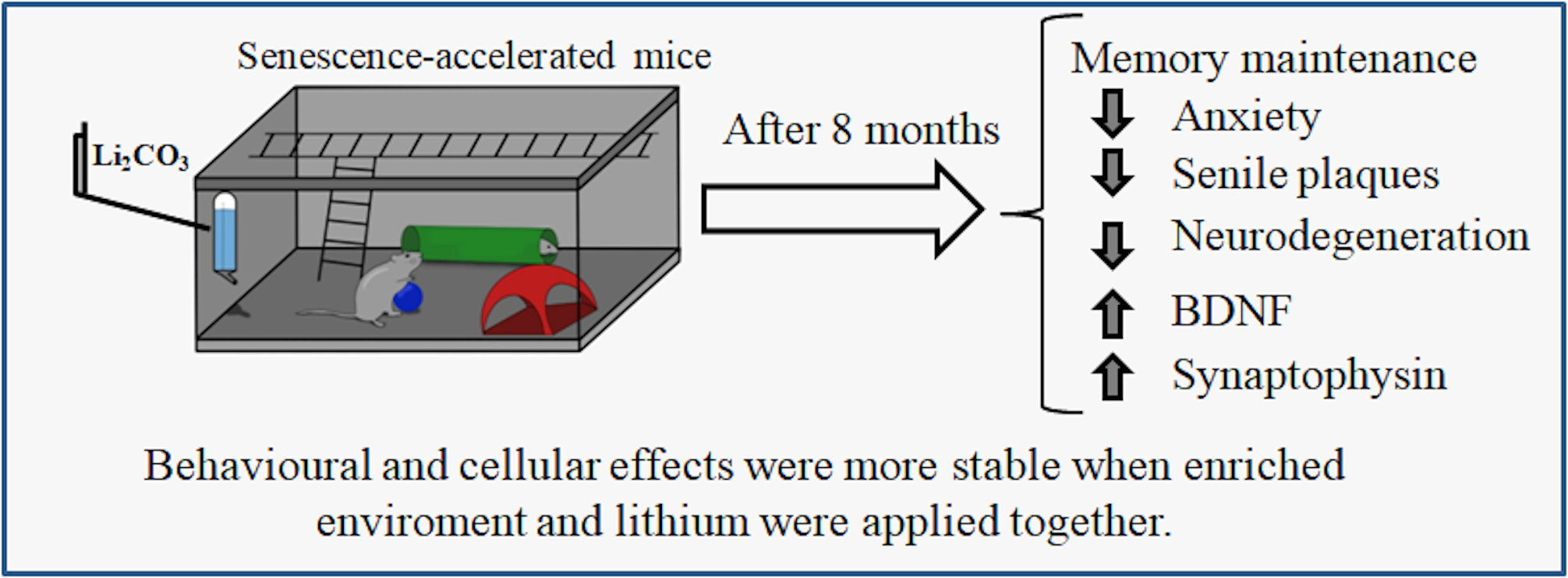

Supplement: Supplementary file 1 [file Image_1.TIF]
